# Human antibody 3E1 targets the HA stem region of H1N1 and H5N6 influenza A viruses

**DOI:** 10.1038/ncomms13577

**Published:** 2016-12-02

**Authors:** Wenshuai Wang, Xiaoyu Sun, Yanbing Li, Jinpeng Su, Zhiyang Ling, Tianlong Zhang, Fang Wang, Hong Zhang, Hualan Chen, Jianping Ding, Bing Sun

**Affiliations:** 1 National Center for Protein Science Shanghai, State Key Laboratory of Molecular Biology, Center for Excellence in Molecular Cell Science, Institute of Biochemistry and Cell Biology, Shanghai Institutes for Biological Sciences and University of Chinese Academy of Sciences, Chinese Academy of Sciences, 320 Yue-Yang Road, Shanghai 200031, China; 2 CAS Key Laboratory of Molecular Virology & Immunology, Institut Pasteur of Shanghai, Chinese Academy of Sciences, University of Chinese Academy of Sciences, 320 Yue-Yang Road, Shanghai 200031, China; 3 State Key Laboratory of Veterinary Biotechnology, Harbin Veterinary Research Institute, Chinese Academy of Agricultural Sciences, Harbin 150001, China; 4 State Key Laboratory of Cell Biology, CAS Center for Excellence in Molecular Cell Science, Institute of Biochemistry and Cell Biology, Chinese Academy of Sciences, 320 Yue-Yang Road, Shanghai 200031, China; 5 Shanghai Science Research Center, Chinese Academy of Sciences, Shanghai 201204, China

## Abstract

As influenza A viruses remain a major threat to human health worldwide, the discovery of broadly neutralizing monoclonal antibodies that recognize conserved epitopes would facilitate the development of antibody-based therapeutic strategies. Here we report that a V_H_4-4-encoded human mAb named 3E1 could neutralize H1 and H5 subtype viruses *in vitro* and protect mice against the H1N1 and H5N6 viruses by inhibiting the low pH-induced conformational rearrangement of haemagglutinin (HA), hence blocking membrane fusion. The crystal structures of 3E1 Fab in complex with HA of two H1N1 strains reveal that 3E1, with both heavy and light chains, binds to a conserved epitope of the HA stem region, comprising parts of the fusion peptide, the F subdomain and the outermost β-strand preceding helix A. Altogether, these data suggest the potential of 3E1 as a therapeutic drug against H1 and H5 subtype viruses.

## Abstract (web summary)

Treatment of influenza A viruses with broadly neutralizing monoclonal antibodies is an area of active research. Here, the authors characterise a human monoclonal antibody called 3E1 that was reactive against both H1 and H5 viruses *in vitro* and demonstrated some treatment efficacy in mice.

## Graphical abstract

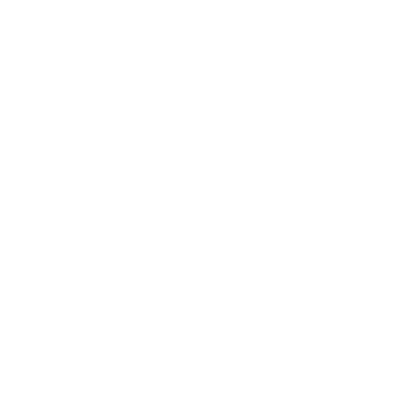

Influenza A virus (IAV), a genus of the Orthomyxoviridae family, remains a grave and persistent threat to human health, while imposing a heavy economic burden on patients and countries worldwide^1^,^2^. With peaks in winter and/or early spring, influenza epidemics recur yearly and cause 3–5 million cases of severe illness and 0.25–0.5 million deaths worldwide annually, particularly among those in high-risk groups, including infants, elders and immune-compromised persons^2^,^3^. In addition to the 2009 swine flu, three influenza pandemics (1918 Spanish, 1957 Asian and 1968 Hong Kong) in the twentieth century killed more than 40 million people^4^. To date, vaccination remains the most effective measure by which to control infection and alleviate the morbidity and mortality of influenza epidemics and pandemics^5^,^6^. However, due to rapid antigen drift, the trivalent vaccines that can elicit antibodies that neutralize vaccine strains and other homologous strains must be updated almost annually based on WHO surveillance and prediction of the strains that will circulate the next year^6^,^7^,^8^. Mismatches between vaccines and circulating strains may aggravate the epidemic^6^,^9^. Moreover, faced with sporadic pandemics resulting from antigen shift, herd immunity cannot be achieved in the short time that exists before an adequate supply of vaccine is available^6^,^7^,^8^. Thus, antiviral drugs are also available to treat influenza in the early stage of infection, mitigating the severity of epidemics and pandemics^5^. Currently, most circulating viruses are resistant to adamantane, which blocks the M2 ion channel, and, therefore, neuraminidase inhibitors are the recommended first-line countermeasures^3^,^10^,^11^. Unfortunately, some new viruses have already evolved to resist neuraminidase inhibitors^11^,^12^. Therefore, the development of more effective antiviral drugs and therapeutic monoclonal antibodies that can provide broad protective activities is urgently needed to prevent and treat influenza.

Haemagglutinin (HA) of IAV is an envelope glycoprotein that is responsible for receptor attachment and membrane fusion, leading to virus invasion^13^. Most antibodies elicited by vaccination or infection mainly target the head region of HA, but the highly variable nature of the epitopes within this region restricts their application to homologous strains^14^. A mouse mAb, C179, can neutralize divergent IAV subtypes *in vitro* and protect mice from infection by passive immunization^15^. Subsequent studies confirmed that C179 targets the highly conserved stem region of HA (ref. 16), indicating that antibodies recognizing the stem region might have higher cross-reactivity. To date, several broadly neutralizing monoclonal antibodies (bnmAbs) targeting epitopes in the HA stem region have been identified by phage display of libraries from non-vaccinated or influenza-vaccinated donors and the screening of plasma cells from influenza-vaccinated or infected donors^17^,^18^,^19^,^20^,^21^,^22^,^23^.

We previously reported a V_H_4-4-encoded mAb isolated from a volunteer who had received the A(H1N1)09pdm vaccine; the mAb, named 3E1, can target the HA stem region^24^. In this work, we demonstrate that 3E1 exhibited broad neutralizing activity against H1 and H5 subtype viruses *in vitro* and protected mice against H1N1 and H5N6 viruses *in vivo* by inhibiting the low pH-induced HA conformational rearrangement, hence blocking membrane fusion. The crystal structures of 3E1 Fab in complex with the HA protein of two H1N1 strains showed that both the heavy and light chains of 3E1 recognized a conserved epitope comprising the C-terminus of the fusion peptide, part of the F subdomain and the C-terminus of the outermost β-strand preceding helix A. Our structural and biological data suggest the potential of 3E1 as a therapeutic drug against H1 and H5 subtype viruses.

## Results

### 3E1 neutralizes H1 and H5 subtype viruses *in vitro*

We previously identified a fully human mAb named 3E1 from the pandemic H1N1 HA-specific memory B cells isolated from the peripheral blood mononuclear cells of a volunteer vaccinated with the A(H1N1)09pdm vaccine; this mAb was shown to have a broad neutralizing activity against several subtypes of IAVs and to target the conserved stem region of HA (ref. 24). The analysis of the 3E1 DNA sequence showed that 3E1 shares 95.43% and 98.11% identity with the IGHV4-4 × 07 and IGKV1-5 × 03 germline genes, respectively. Consistently, 3E1 harboured seven somatic mutations in the VH amino acid sequence and two in VL, compared with the unmutated common ancestor, indicating 3E1 is less hypermutated (Supplementary Fig. 1). To date, several bnmAbs against IAVs have been characterized, including FI6v3, CR9114, CR6261, F10 and Fab 3.1, all of which bind to the stem region of HA. CR9114, CR6261 and F10 are derived from the V_H_1-69 family and only bind to HA through the heavy chain^17^,^20^,^21^. Additionally, though they carry a high load of somatic mutations, with the germline-encoded F45 and Y98, a single mutation in HCDR1 or HCDR2 loops is required for the development of bnmAbs (ref. 25). FI6v3 and Fab 3.1 originate from the V_H_3-30 family and bind to HA through both heavy and light chains^18^,^23^. With fewer hypermutations, 3E1, which is encoded by the V_H_4-4 germline gene, may target the stem region of HA differently from the previously reported bnmAbs.

**Figure 1:**
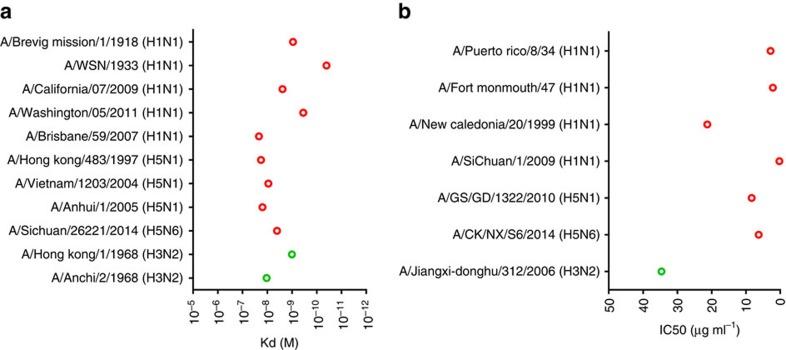
*In vitro* binding and neutralization activity of 3E1 mAb. (**a**) Binding affinity (Kd) of 3E1 mAb to the HAs of various strains of H1, H5 and H3 subtype IAVs. Data represent a representative experiment from three independent experiments. (**b**) *In vitro* microneutralization (IC_50_) of 3E1 mAb against various strains of H1, H5 and H3 subtype IAVs. Graphs used for IC_50_ values determined by averaging neutralization titre of three independent experiments.

Our preliminary neutralization assay showed that 3E1 could neutralize the SC09 H1N1 virus. As 3E1 targets the stem region of HA, which is recognized by bnmAbs, we assumed that 3E1 might have neutralizing activity against other IAV strains. To verify the cross-reactive breadth of 3E1, we determined the binding affinities of 3E1 to a panel of recombinant HAs from group 1 IAVs. The data showed that 3E1 bound to HAs from widely divergent strains of H1 and H5 subtype viruses, and two strains of H3 subtype viruses with high affinity (K_d_ of 10–10^−2^ nM; Fig. 1a).

To confirm the neutralizing potency of 3E1, divergent IAV strains were evaluated using an *in vitro* microneutralization assay. Consistent with the binding assay, 3E1 neutralized several H1N1 strains with an IC_50_ of 0–20 μg ml^−1^ (Fig. 1b). Similarly, 3E1 also effectively neutralized two highly pathogenic avian influenza strains, H5N1 and H5N6, with comparable IC_50_ values. Interestingly, 3E1 also exhibited the ability to neutralize an H3N2 strain with a relatively higher IC_50_ value (∼30 μg ml^−1^; Fig. 1b). These results suggest that 3E1 has a broad neutralizing activity against divergent IAV strains of the H1 and H5 subtypes.

### 3E1 protects mice against H1N1 and H5N6 viruses *in vivo*

On the basis of high-level cross-neutralizing activity of 3E1 against group 1 viruses (Fig. 1b), we chose A/Sichuan/2009(H1N1) and A/CK/NX/S6/2014(H5N6), two representatives of group 1 IAVs, for prophylactic and therapeutic assays in the BALB/c mouse challenge model. A/Sichuan/2009(H1N1) is a pdm09 strain that circulated during the 2009 pandemic, and A/CK/NX/S6/2014(H5N6) is a highly pathogenic strain that recently caused severe infections in humans in China.

**Figure 2:**
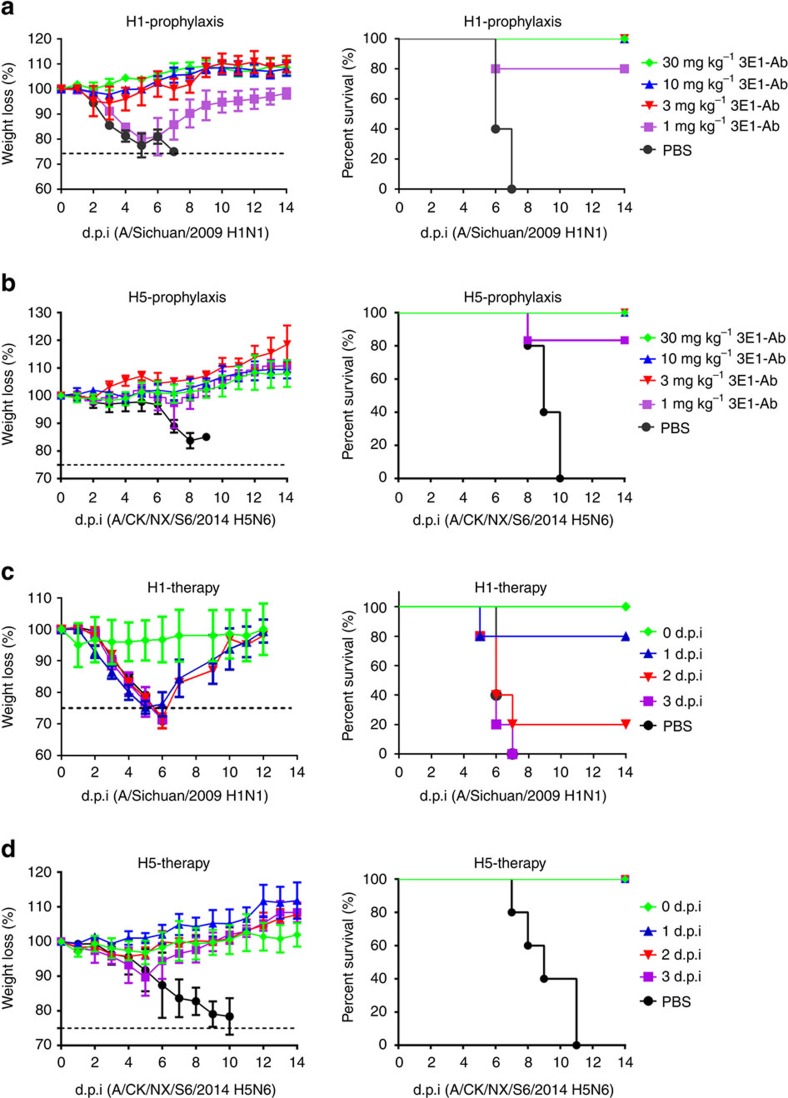
Prophylactic and therapeutic efficacy of 3E1 mAb in mice. (**a**,**b**) Prophylactic efficacy of 3E1 mAb against a lethal challenge with the A/Sichuan/09/2009(H1N1) virus (**a**) or A/CK/NX/S6/2014(H5N6) virus (**b**). The weight loss (left) and survival curves (right) of mice treated with 30, 10, 3 or 1 mg kg^−1^ of 3E1 mAb or PBS buffer 24 h before lethal challenge by an intranasal inoculation with the H1-SC09 or H5-NX14 virus (at day 0) are shown. (**c**,**d**) Therapeutic efficacy of 3E1 mAb against a lethal challenge with the A/Sichuan/09/2009(H1N1) (**c**) or A/CK/NX/S6/2014(H5N6) virus (**d**). The weight loss (left) and survival curves (right) of mice treated with PBS buffer (at day 1) or 20 mg kg^−1^ of 3E1 mAb right after or 1, 2, 3 days after lethal challenge by an intranasal inoculation with the H1-SC09 or H5-NX14 virus (at day 0) are shown. Error bars represent mean±s.d. Data represent a representative experiment from three independent experiments.

In the prophylaxis assay, the mice were first administered different doses of 3E1 (30, 10, 3 or 1 mg kg^−1^), and challenged with a lethal dose of the H1N1 or H5N6 virus 24 h later. The administration of a high dose of 3E1 (≥3 mg kg^−1^) fully protected the mice from H1N1 infection, with a slightly increased body weight over 2 weeks (Fig. 2a). After the administration of a low dose of 3E1 (1 mg kg^−1^), the mice were also highly protected from H1N1 infection, with an 80% survival rate (Fig. 2a). Using the highly pathogenic H5N6 virus, the mice were also fully protected from infection at high doses of 3E1 (≥3 mg kg^−1^), and the administration of the lowest dose of 3E1 (1 mg kg^−1^) also protected the mice with an 80% survival rate and a slightly increased body weight over 2 weeks (Fig. 2b).

In the therapeutic assay, the mice were first challenged with a lethal dose of the H1N1 or H5N6 virus and then administered a fixed dose of 3E1 (20 mg kg^−1^) on different days (0, 1, 2 or 3 days). 3E1 treatment on day 1 significantly protected the mice against H1N1, with an 80% survival rate; the body weight was reduced but then gradually recovered over the next 5 days (Fig. 2c). However, 3E1 treatment on day 2 only moderately protected the mice, with a 20% survival rate, and 3E1 treatment on day 3 offered no protection to the mice (Fig. 2c). Surprisingly, 3E1 treatment fully protected the mice against H5N6, even when administered on day 3 (Fig. 2d). Moreover, following a challenge with H5N6 and subsequent treatments with 3E1, the body weights of the mice fluctuated less than those with H1N1 infection over 2 weeks (Fig. 2c,d). Taken together, the prophylactic and therapeutic experiments indicate that 3E1 could protect mice against the H1N1 and H5N6 viruses.

### Crystal structures of the 3E1 Fab-HA complexes

To elucidate the structural basis of the broad neutralizing activity of 3E1 against group 1 IAVs, we determined the crystal structures of 3E1 Fab in complex with the HA ectodomain of two H1N1 strains, namely A/California/04/2009 (CA09) and A/Washington/05/2011 (WA11), at 2.9 and 3.1 Å resolution, respectively (Table 1). CA09 and WA11 are two pandemic and reassorted group 1 IAVs, which differ by eight residues in their HA extracellular domains. Like the HA proteins of most IAVs, the HA of WA11 exists as a stable trimer in solution. However, the trimeric HA of CA09 is unstable in solution and tends to dissociate into monomers^26^; thus, we introduced two mutations (G205C and R220C) that form a disulfide bond at the monomer–monomer interface, leading to the formation of a stable HA trimer^27^ (Supplementary Fig. 2).

**Figure 3:**
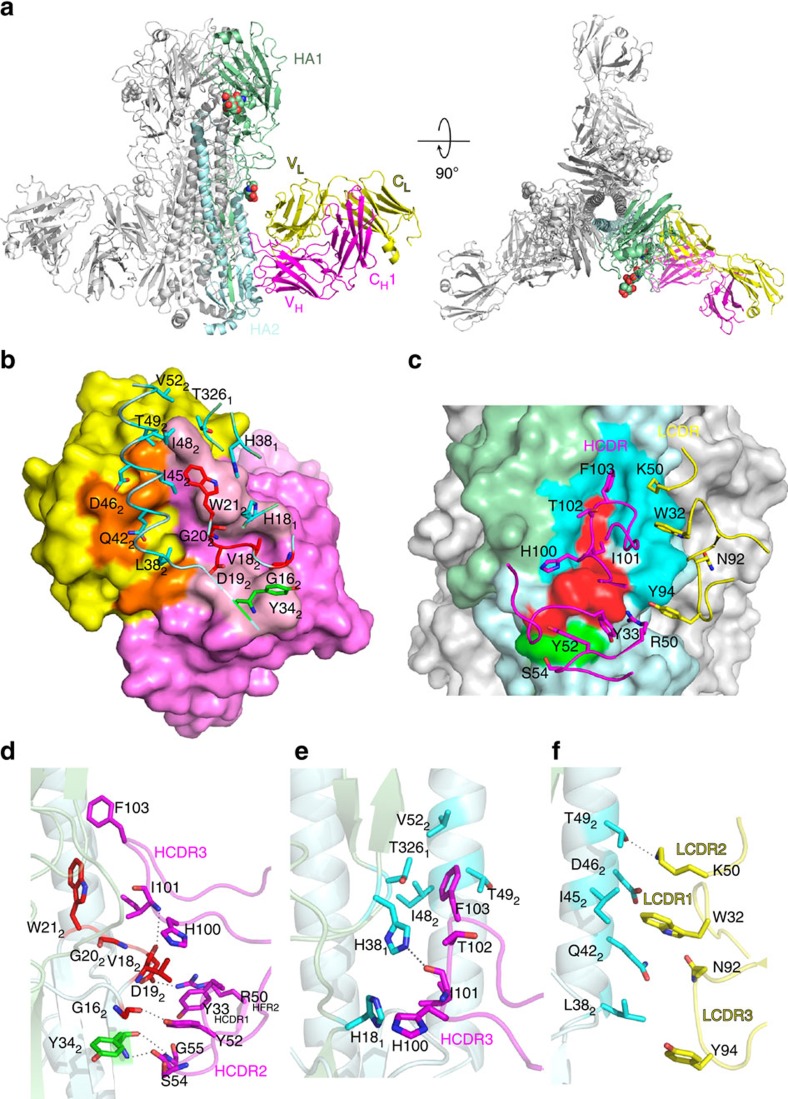
Structure of the 3E1 Fab–CA09 HA complex. (**a**) The overall structure of the 3E1 Fab–CA09 HA complex is shown in cartoon representation from the side view and top view. HA1 and HA2 are coloured in pale green and pale cyan, respectively. The heavy chain and light chain of 3E1 Fab are coloured in magenta and yellow, respectively. The glycans are shown with spheres. 3E1 Fab binds to the stem region of HA. (**b**) The interaction interface between 3E1 Fab and HA looking down at the Fab (paratope). The Fab is shown in surface representation, with heavy chain in violet, and the HA in cartoon. The residues of the heavy chain and light chain at the antigen-binding site coloured in light pink and orange, respectively. The key residues of the epitope are shown with side chains: these of HA1 are denoted with a subscript 1 and these of HA2 with a subscript 2, and these of the fusion peptide are coloured in red, these of the F subdomain in cyan, and these of the β-strand in green. (**c**) The interaction interface between 3E1 Fab and HA looking down at the HA (epitope). The HA is shown in surface representation and the Fab in cartoon. The key residues of the antigen-binding site are shown with side chains. (**d**–**f**) Specific interactions between the fusion peptide (red) and β-strand (green) and the HCDR loops (magenta) (**d**), between the F subdomain (cyan) and the HCDR3 loop (magenta) (**e**) and between the F subdomain (cyan) and the LCDR loops (yellow) (**f**). The key residues are shown with side chains.

The structures of the 3E1 Fab–HA complexes were solved by the molecular replacement method. There is one Fab-HA complex in the asymmetric unit, and three Fab–HA complexes related by 3-fold crystallographic symmetry form a trimer (Fig. 3a and Supplementary Fig. 3). 3E1 Fab consists of the heavy chain, which folds into the V_H_ and C_H_1 domains, and the light chain, which folds into the V_L_ and C_L_ domains (Fig. 3a). The HA protein is composed of the HA1 and HA2 polypeptide chains, which fold into the head and stem regions. In both complexes, most of the polypeptide chains are well defined, with the exception of a few surface-exposed loops (residues 80–81, 203, 272 of CA09 HA1, residues 1–3, 8 of CA09 HA2, residues 151, 157, 182–184 of the light chain and residues 42, 135–142, 199 of the heavy chain of 3E1 in the 3E1 Fab–CA09 HA complex; residues 271–272 of WA11 HA1, residues 1–4 of WA11 HA2, residues 181 of the light chain and residues 41, 135–142, 199 of the heavy chain of 3E1 Fab in the 3E1 Fab–WA11 HA complex). Additionally, we were able to build two sugar residues at Asn97 and one sugar residue at Asn286 of CA09 HA, and two sugar residues at Asn97 of WA11 HA. A comparison of the structures of the two complexes did not reveal notable differences in the overall structure, indicating that the introduction of the disulfide bond in the CA09 HA trimer has no effect on the overall conformation of the HA trimer. Thus, we used the 3E1 Fab–CA09 HA complex in the subsequent structural analysis, unless otherwise specified.

### Interactions between 3E1 Fab and HA

In the 3E1 Fab–CA09 HA complex, 3E1 Fab specifically recognizes and binds to the conserved stem region of HA (Fig. 3a). Although the two proteins have a shape complementarity value of 0.59, which is slightly smaller than the average value (0.64–0.68) of the antigen–antibody complexes^28^, the interacting interface buries a total solvent accessible surface area of 1597 Å^2^ (821 Å^2^ for Fab and 776 Å^2^ for HA), which is no less than the average value of the antigen–antibody complexes (1,200–1,700 Å^2^ for total, 600–900 Å^2^ for Fab, and 560–850 Å^2^ for antigen)^29^. Among the buried surface area of the Fab, the heavy chain contributes 78% and the light chain 22%, consistent with the notion that the heavy chain typically contributes more than the light chain^30^ (Fig. 3b). The antigen-binding site or paratope of 3E1 is composed of large portions of the three HCDR loops and small portions of the three LCDR loops and HFR2 (Fig. 3b,c). The HA epitope consists of several discontinuous segments, including the C-terminus of the fusion peptide (residues 16–21 of HA2), part of the F subdomain (residues 38–52 of HA2 and residues 18, 38 and 326 of HA1) and the C-terminus of the outermost β-strand preceding helix A (residue 34 of HA2; Fig. 3b,c).

At the centre of the interacting interface, the three HCDR loops and the HFR2 of 3E1 Fab form a shallow cleft to accommodate the C-terminus of the HA2 fusion peptide via both hydrophilic and hydrophobic interactions (Fig. 3c,d). Specifically, the side chain of Tyr52 of HCDR2 forms a hydrogen bond with the main-chain carbonyl of Gly16 of the fusion peptide, the main-chain amine of Ile101 of HCDR3 forms a hydrogen bond with the main-chain carbonyl of Val18 of the fusion peptide, and the side chain of Arg50 of HFR2 forms a salt bridge with the side chain of Asp19 of the fusion peptide (Fig. 3d). Additionally, Tyr33 of HCDR1 makes hydrophobic contacts with Val18 and Asp19 of the fusion peptide, Tyr52 of HCDR2 makes hydrophobic contacts with Val18 of the fusion peptide, and His100, Ile101 and Phe103 of HCDR3 form extensive hydrophobic interactions with Val18, Asp19, Gly20 and Trp21 of the fusion peptide (Fig. 3d).

At the upper part of the interacting interface, the HCDR3 loop and the three LCDR loops mainly interact with the F subdomain via hydrophobic interactions (Fig. 3e,f). Specifically, His100, Ile101, Thr102 and Phe103 of HCDR3 form extensive hydrophobic interactions with His18, His38 and Thr326 of HA1, and Ile48, Thr49 and Val52 of helix A of HA2, respectively (Fig. 3e). Moreover, the main-chain carbonyl of Ile101 of HCDR3 forms a hydrogen bond with the side chain of His18 of HA1 (Fig. 3e). The LCDR loops lie on top and form extensive hydrophobic contacts with helix A (Fig. 3f). Trp32 of LCDR1 forms extensive hydrophobic contacts with Gln42, Ile45 and Asp46 of helix A; Lys50 of LCDR2 forms hydrophobic contacts with Ile45, Asp46 and Thr49 of helix A, and Asn92 and Tyr94 of LCDR3 form hydrophobic contacts with Gln42 and Leu38 of helix A (Fig. 3f). Moreover, the side chain of Lys50 of LCDR2 forms a hydrogen bond with the side chain of Thr49 of helix A (Fig. 3f).

At the lower part of the interacting interface, the HCDR2 loop interacts with the C-terminus of the outermost β-strand preceding helix A via two hydrogen bonds, including the interaction between the main-chain carbonyl of Ser54 of HCDR2 and the main-chain amine of Tyr34 of the β-strand and the interaction between the main-chain amine of Gly55 of HCDR2 and the carbonyl of Tyr34 of the β-strand (Fig. 3d). In addition, the amino acid substitutions of 3E1 are not involved in the formation of the paratope, thus do not interact with the amino acids in the epitope, indicating these substitutions are not essential (Supplementary Fig. 1). Taken together, our structural data show that 3E1 Fab primarily uses the six CDR loops to recognize and bind to a conformational epitope of the stem region of HA largely via hydrophobic interactions.

### 3E1 recognizes a unique conformational epitope of HA

**Figure 4:**
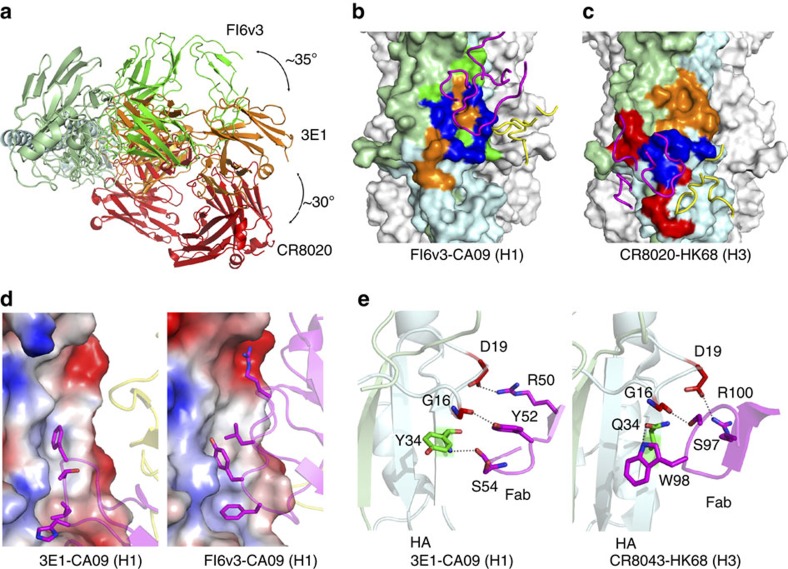
Binding specificities of 3E1 compared with the type I and type II bnmAbs. (**a**) Top view overlay of the crystal structures of the 3E1 (orange), FI6v3 (green, type I bnmAb) and CR8020 (red, type II bnmAb) Fabs in complexes with HA. For clarity, only the HA in the 3E1 Fab–CA09 HA complex is shown. The three Fabs bind to HA from different orientations. (**b**) Comparison of the epitopes between 3E1 and FI6v3. (**c**) Comparison of the epitopes between 3E1 and CR8020. The HAs in the FI6v3 Fab-CA09 HA complex (PDB code: 3ZTN) and CR8020 Fab-HK68 HA complex (PDB code: 3SDY) are shown in surface representation and the CDRs of FI6v3 and CR8020 in cartoons. The epitopes of 3E1, FI6v3 and CR8020 are coloured in orange, green and red, respectively, and the overlapping regions are coloured in blue. (**d**) Similar to FI6v3 (type I bnmAbs), 3E1 Fab makes hydrophobic contacts with the F subdomain of HA. The HAs are shown as electrostatic surface representations and the Fabs as cartoons in the same orientation. (**e**) Similar to CR8043 (type II bnmAb), 3E1 makes hydrophilic interactions with the fusion peptide of HA. The residues of HAs and Fabs involved in the interactions are shown with side chains.

To date, the crystal structures of several bnmAbs in complexes with their specific HAs have been reported. Although these bnmAbs all target the conserved stem region of HA, they recognize different epitopes and thus could be divided into two types: type I bnmAbs (FI6v3, CR9114, CR6261, F10, Fab3.1 and C179) recognize the F subdomain of HA; and type II bnmAbs (CR8020 and CR8043) recognize the fusion peptide and the outermost β-strand preceding helix A of HA (refs 16, 17, 18, 19, 20, 21, 22, 23) (Supplementary Fig. 4a,b). Consequently, the binding orientations of the two types of bnmAbs to HA differ by approximately 65° (refs 16, 17, 18, 19, 20, 21, 22, 23; Supplementary Fig. 4c). Intriguingly, 3E1 recognizes a conformational epitope consisting of the C-terminus of the fusion peptide, part of the F subdomain, and the C-terminus of the outermost β-strand preceding helix A (Fig. 3c), which constitute the major parts of the epitopes recognized by both types I and II bnmAbs (Fig. 4a–c). Specifically, the epitope (∼776 Å^2^) recognized by 3E1 overlaps with 488 Å^2^ out of the 760 Å^2^ of the epitope recognized by FI6v3 (a typical type I bnmAb) and 460 Å^2^ out of the 804 Å^2^ of the epitope recognized by CR8020 (a typical type II bnmAb; Fig. 4b,c). Consistently, the binding orientation of 3E1 Fab to HA lies exactly between those of types I and II bnmAbs: an ∼35° rotation clockwise from that of type I bnmAbs and approximately 30° rotation anti-clockwise from that of type II bnmAbs (Fig. 4a). Furthermore, the interactions of 3E1 with HA combine most of the interactions of both types I and II bnmAbs with their HAs. Similar to type I bnmAbs, 3E1 makes extensive hydrophobic contacts with residues of the F subdomain of HA via large hydrophobic and aromatic residues (Fig. 4d and Supplementary Fig. 5); and similar to type II bnmAbs, 3E1 makes hydrophilic interactions with residues of the fusion peptide and the β-strand (Fig. 4e). Altogether, these results indicate that the epitope of 3E1 is comprised of the major parts of the epitopes recognized by both types I and II bnmAbs, and thus represents a unique epitope.

### The 3E1 epitope is highly conserved in the H1 and H5 viruses

A comparison of the HA sequences from divergent strains of group 1 IAVs shows that the residues composing the epitope of 3E1 are highly conserved, particularly in the HAs of the H1 and H5 subtypes (Table 2), which is consistent with the high-level binding and neutralizing activity of 3E1 against H1 and H5 subtype viruses (Fig. 1a,b). One exception is that the H1 subtype IAVs strongly prefer to have a Leu or Gln and the H5 subtype IAVs prefer a Lys at position 38 of HA2. However, as Leu38 in helix A of HA2 makes only one hydrophobic contact with 3E1 Fab, as shown in the structures, the replacement of Lys38 in H5 HAs may not be able to attenuate the interactions between the HAs and 3E1 Fab.

**Figure 5:**
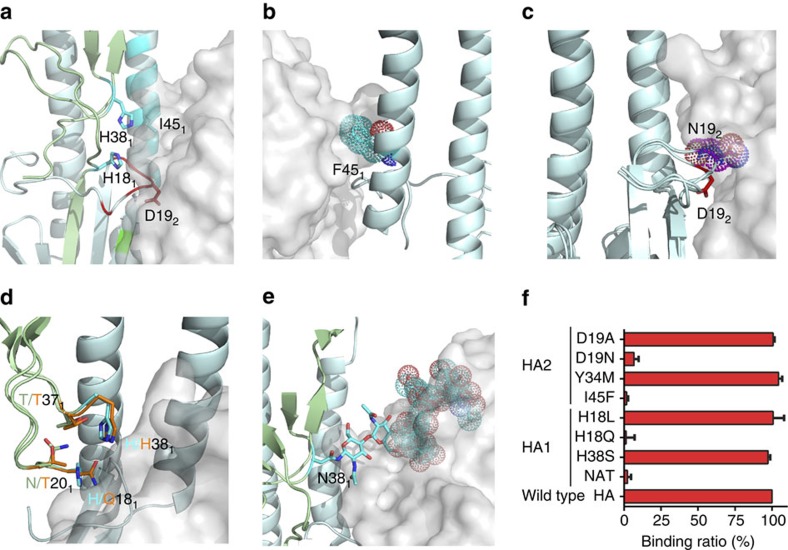
Residues in the natural variants that may affect 3E1 recognition. (**a**) Locations of the key residues of HA (shown with side chains) that may affect 3E1 recognition. The HA is shown in cartoon and the 3E1 Fab in surface representation. The colour coding of HA is the same as Fig. 3b. (**b**) The I45F variant (shown with side chain and dots) in HAs of the H2 subtype viruses could cause steric hindrance with the HCDR3 loop of 3E1 in the HA of Jap57 (H2) structure (PDB code: 2WRD). (**c**) The D19N variant in HAs of the H13 subtype viruses adopts a different side-chain conformation in the HA of ML77 (H13) structure (coloured in magenta, PDB code: 4KPQ), which could cause steric hindrance with 3E1. (**d**) The H18Q variant in HAs of the H9 subtype viruses makes relatively weaker interactions with Asn20, Thr37 and His38 in the HA of KO98 (H9) structure (coloured in orange, PDB code: 1JSD), which might interfere with the recognition by 3E1. (**e**) The glycans at Asn38 in the HAs of the H3 and H7 subtype viruses might obscure the recognition by 3E1. The glycans at Asn38 in the HA of HK68 (H3) structure (PDB code: 4WE4) are shown as cyan sticks. (**f**) Relative binding ratio of the mutants of WA11 HA with the 3E1 Fab. Bars represent mean±s.d. Data represent a representative experiment from three independent experiments.

Although our biochemical data show that 3E1 bound to HAs from widely divergent strains of H1 and H5 subtype viruses and two of the four tested H3 subtype viruses (Fig. 1a). The sequence alignment shows that the HAs of those strains have some variant residues in the C-terminus of the fusion peptide or the F subdomain, which may contribute to their inability to bind to 3E1 (Table 2 and Fig. 5a). To investigate this possibility, we constructed a series of mutations in WA11 HA and measured the relative binding affinities of these mutants with 3E1.

In summary, according to the mutation binding assay results and related structural analysis, 3E1 should overcome at least four obstacles to achieve a broader neutralizing activity against divergent IAVs. First, the H1 subtype viruses have a strictly conserved Ile45 in helix A of HA2, which is substituted with a strictly conserved Phe in the H2 subtype viruses (Table 2). In the structure of H2N2 HA, Phe45 of HA2 caused steric hindrance with the HCDR3 loop of 3E1 (Fig. 5b). The biochemical data consistently show that the I45F mutation abolished the binding with 3E1 (Fig. 5f). These results explain why 3E1 does not bind to the HAs of the H2 subtype viruses. Second, the H1 subtype viruses have a strictly conserved Asp19 in the fusion peptide of HA2, which is substituted with a strictly conserved Asn in the H13 and H16 subtype viruses (Table 2). In contrast to the H1N1 HA, in the structure of the H13N6 HA, Asn19 of HA2 adopted a different side-chain conformation, which caused steric hindrance with 3E1 Fab, even though Asp and Asn are very similar (Fig. 5c). The D19N mutation consistently caused a significant reduction in binding with 3E1 (Fig. 5f). These results explain the reduced binding of 3E1 to the HAs of the H13 and H16 subtype viruses. Third, the H1 subtype viruses have a strictly conserved His18 in HA1, which is located near the C-terminus of the fusion peptide; this residue is substituted with a strictly conserved Gln in the H9 subtype viruses (Table 2). The replacement of His18 in HA1 with Gln might attenuate its interactions with Asn20, Thr37 and His38 in HA1 (Fig. 5d). Apparently, the H18Q mutation in HA1 abolished the binding with 3E1 (Fig. 5f), explaining why 3E1 did not bind to the H9 subtype viruses. Fourth, the H1 subtype viruses have a strictly conserved His38 in HA1, which is substituted with a strictly conserved Asn in the H3 and H7 subtype viruses. The predominant conformation of the glycans linked to Asn38 in the HAs of the H3 and H7 subtype viruses obscured the epitope recognized by 3E1 (Fig. 5e). Consistently, on introduction of this glycosylation site in WA11 HA, the mutant also abolished the binding with 3E1 (Fig. 5f). These results partially explain why 3E1 only bound to two of the tested H3 HAs, as well as the relatively higher IC_50_ against one H3 strain. Thus, in addition to the conserved epitopes among divergent IAVs, some residues in the variants also play a role in the interactions with 3E1, which provide sufficient data to engineer antibodies based on 3E1.

Taken altogether, these results indicate that the epitope of 3E1 is highly conserved in the H1 and H5 subtype viruses, which agrees well with the *in vivo* experiments, showing that 3E1 could protect mice from H1N1 and H5N6 infection.

### 3E1 inhibits HA conformational change

**Figure 6:**
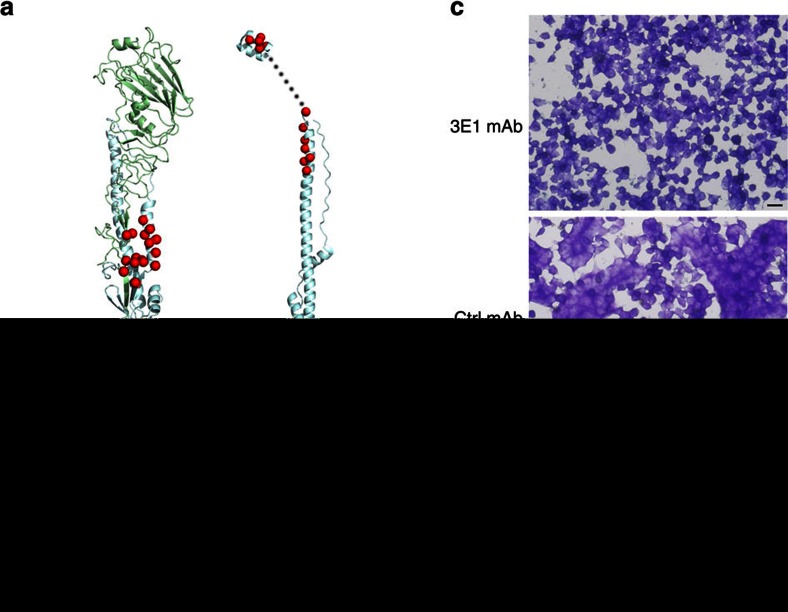
3E1 inhibits low-pH-induced HA conformational change. (**a**) Location of the residues forming the 3E1 epitope in the pre-fusion-state monomeric HA (left panel; PDB code: 3LZG) and post-fusion-state monomeric HA (right panel; PDB codes: 1QU1 and 2KXA). The residues of the epitope are shown as red spheres. (**b**) Protease sensitivity assay of HA in the presence of 3E1 mAb. Exposure of HA to low pH converts the HA to the protease-susceptible, post-fusion state (lanes 3, 7). Treatment of HA with 3E1 mAb before low-pH treatment, but not the control mAb, blocks the pH-induced conformational change, retaining HA in the protease-resistant, pre-fusion state (lanes 4, 8). Data represent a representative experiment from three independent experiments. (**c**) 3E1 mAb blocks trypsin-activated, low pH-triggered, HA-mediated cell–cell fusion. HEK-293 T cells were transfected with full-length WA11 HA and treated with trypsin, and then 3E1 or control mAb was added followed by low-pH treatment. Treatment of 3E1 mAb could inhibit the formation of syncytium (upper panel), while the control mAb could not (middle panel). Scale bar represents 20 μm. Data represent a representative experiment from three independent experiments.

Unlike most antibodies that target the head region of HA, which blocks virus attachment, 3E1 recognizes an epitope located in the stem region of HA. On exposure to low pH, the stem region of HA undergoes substantial conformational change, transforming from the pre-fusion state to the post-fusion state and bringing the viral membrane and the target endosomal membrane into close proximity to trigger membrane fusion^31^,^32^ (Fig. 6a). Thus, like the other bnmAbs that target the stem region of HA, 3E1 is thought to inhibit the low pH-induced conformational rearrangement, thus blocking membrane fusion and virus invasion^18^,^19^,^33^. In our protease sensitivity assay, the post-fusion HA is highly sensitive to proteases and could be degraded by trypsin (Fig. 6b, lanes 3, 7). In contrast, when it was incubated with 3E1 before acidification, the activated HA became protease-resistant (Fig. 6b, lane 4, 8), suggesting that the binding of 3E1 prevents the conformational change of the activated HA from the pre-fusion state to the post-fusion state. Additional evidence showing that 3E1 acts to inhibit the conformational change is illustrated by the remarkably efficient inhibition of syncytia formation in HEK-293 T cells expressing full-length HA0 at the low pH in the presence of 3E1 (Fig. 6c). In summary, by blocking HA in the pre-fusion state at low pH, 3E1 inhibits membrane fusion and hence successfully interferes with virus infectivity.

## Discussion

Currently, the increasingly frequent outbreaks of novel subtypes of zoonotic influenza viruses from domestic animals are becoming a major challenge for preventing and treating influenza^34^. The identification of several fully human bnmAbs targeting the conserved stem region of HA has provided insights into antibody-based therapy for severe and late-stage influenza, particularly for infants and the elderly^35^. The characteristics of the epitopes and paratopes elicited by the structures of these Fab-HA complexes could be employed for the rational design of more effective antiviral drugs and universal vaccines^35^.

In this work, we performed structural and functional studies of a fully human mAb named 3E1 and found that 3E1 exhibits a reactive profile against a wide range of H1 and H5 subtype viruses. Specifically, 3E1 could neutralize the widely divergent H1 and H5 subtype viruses *in vitro* and protect mice challenged with the H1N1 and H5N6 viruses *in vivo*. The structures of the 3E1 Fab–HA complexes revealed that 3E1 targets a highly conserved epitope comprised of the major parts of the epitopes recognized by both types I and II bnmAbs (refs 16, 17, 18, 19, 20, 21, 22, 23). In addition, the binding orientation of 3E1 Fab with HA is located in between those of the types I and II bnmAbs, and the interactions of 3E1 Fab with HA combine the featured interactions of both types I and II bnmAbs, thus representing a distinct bnmAb–HA interaction pattern^16^,^18^,^20^,^22^,^23^. Altogether, our structural and functional data demonstrate that 3E1 is a unique bnmAb against H1 and H5 subtype viruses.

As H1N1 is one of the three prevalent virus subtypes in annual epidemics and the highly pathogenic H5 avian influenza viruses (H5N1 and H5N6) continue to be a hazard for human populations^3^,^36^, 3E1 could be an ideal candidate for developing antibody-based therapies against current and future circulating influenza viruses. In addition, it was previously suggested that the glycans on Asn21 in the HAs of group 1 IAVs and the glycans on Asn38 in the HAs of group 2 IAVs are responsible for the limited neutralizing ability of group 1 bnmAbs against group 2 viruses and *vice versa*^19^,^20^. As the HCDR1 loop of 3E1 exhibits restricted binding to group 1 HAs due to the Asn21 glycans, the HCDR1 loop could be engineered to recognize the fusion peptide of group 2 HAs (without Asn21), which might extend its spectrum of recognition. Furthermore, although more convincing experiments are required, the key variant residues identified in the structural and mutational analyses provide the starting point for antibody engineering based on 3E1.

As the fusion peptide of HA constitutes the centre of the epitope targeted by 3E1, it might be advantageous to design polypeptides that can mimic the shallow cleft of the antigen-binding site of 3E1 and thus bind to the fusion peptide of HA, analogous to the design of HB36.3 targeting the F subdomain of HA of group 1 IAVs (ref. 37). Moreover, as a booming field, structural vaccinology utilizes the conserved epitopes of bnmAbs to design immunogens that could induce a broad coverage antibody response in the proof-of-concept tests^38^. Thus, the unique turn of the C-terminus of the fusion peptide that is recognized by 3E1 could provide a blueprint for universal vaccine design^39^. In addition, in contrast to most of the previously reported bnmAbs that are derived from V_H_1-69 germline genes and possess hypermutations^25^, 3E1 is derived from the V_H_4-4 germline gene, which comprises ∼2% of the human antibody repertoire, and the few mutations possessed by 3E1 are all not involved in antigen recognition^40^. In the absence of a high hypermutation rate, vaccines based on the unique epitope targeted by 3E1 might easily achieve active immunization. In summary, the fully human mAb 3E1 itself is of high potency and thus could serve as a potential antiviral drug against H1 and H5 subtype viruses; and additionally the unique epitope targeted by 3E1 sheds some lights on the design of more effective antiviral drugs and potential universal vaccines against IAVs.

## Methods

No statistical methods were used to predetermine sample size. The experiments were not randomized and the investigators were not blinded to allocation during experiments and outcome assessment.

### Viruses

The wild-type influenza viruses A/Fort Monmouth/47(H1N1), A/New Caledonia/20/1999(H1N1), A/Sichuan/1/2009(H1N1) and A/Jiangxi-Donghu/312/2006(H3N2) were kindly provided by Prof Yuelong Shu from the Chinese Center for Disease Control and Prevention, Beijing. The recombinant influenza virus A/Puerto Rico/8/34(H1N1) was rescued by reverse genetics from eight viral genes in pHW2000 plasmids (kindly provided by Robert G. Webster from the St Jude Children’s Research Hospital, University of Tennessee). These viruses were all grown on Madin–Darby canine kidney (MDCK) cells (American Type Culture Collection (ATCC)). The wild-type influenza viruses A/GS/GD/1322/2010(H5N1) and A/CK/NX/S6/2014(H5N6) were grown in eggs. These viruses were used in the *in vitro* microneutralization assay. The A/Sichuan/1/2009(H1N1) and A/CK/NX/S6/2014(H5N6) viruses were used in the *in vivo* assay.

### Preparation of 3E1 Fab

The gene fragments encoding the variable domains of the heavy chain (*V*_H_, 1–121) and light chain (*V*_L_, 1–107) of human mAb 3E1 were cloned into the IgG1 heavy and light expression vectors, AbVec-hIgG1 and AbVec-hIgKappa2, respectively. HEK-293 T cells (ATCC) were transfected with the IgG1 expression plasmids and the expressed antibody was purified from serum-free culture supernatants using Protein G-Agarose beads. 3E1 Fab was obtained by digesting 3E1 mAb with papain (40:1 mAb:papain ratio) for 6 h at 37 °C and purified by ion exchange chromatography using a mono Q anion exchange column (GE Healthcare), followed by size exclusion chromatography using a Superdex 200 10/300 column (GE Healthcare). The purified 3E1 Fab was concentrated to approximately 20 mg ml^−1^ in storage buffer (20 mM HEPES, pH 8.0, and 50 mM NaCl) for use in the structural studies.

### Preparation of the recombinant haemagglutinin proteins

The gene fragments encoding the ectodomains of the HA proteins (residues 11–337 of HA1 and residues 1–176 of HA2, according to the H3 numbering convention) from the A/California/04/2009(H1N1) (CA09) and A/Washington/05/2011(H1N1) (WA11) strains were individually cloned into the baculovirus transfer vector pFastBac1 (Invitrogen) by incorporating a GP67 signal peptide for HA secretion at the N-terminus and a thrombin digestion site, a trimerization Foldon domain^41^, and a 6 × His tag at the C-terminus. To stabilize the HA trimer of CA09, two mutations (G205C and R220C) were introduced into the coding sequence to form a disulfide bond at the monomer-monomer interface^14^. The transfection and virus amplification were performed according to the user manual of the Bac-to-Bac Baculovirus Expression System (Invitrogen). HA was expressed by infecting the suspended Sf9 cells (Invitrogen) for 3 days and recovered from the culture supernatants by metal affinity chromatography using a His trap excel column (GE, EDTA-resistant). The purified HA was subjected to thrombin digestion (GE, a maximum of 50 units per mg of HA0) at room temperature for 20 h to remove the C-terminal Foldon domain and 6 × His tag. The HA0 was further purified by size exclusion chromatography using a Superdex 200 10/300 column and then concentrated to approximately 10 mg ml^−1^ in storage buffer (20 mM HEPES, pH 8.0, and 50 mM NaCl) for use in the structural study.

The HAs from the A/Brevig Mission/1/1918(H1N1), A/WSN/1933(H1N1), A/Brisbane/59/2007(H1N1), A/California/07/2009(H1N1), A/Canada/720/2005(H2N2), A/Hong Kong/483/1997(H5N1), A/Vietnam/1203/2004(H5N1), A/Anhui/1/2005(H5N1), A/Sichuan/26221/2014(H5N6), A/Hong Kong/1073/1999(H9N2), A/black-headed gull/Netherlands/1/00(H13N8), A/black-headed gull/Sweden/5/99(H16N3), A/Hong Kong/1/1968(H3N2), A/Aichi/2/1968(H3N2), A/Brisbane/10/2007(H3N2), A/Taiwan/70113/2007(H3N2), A/Netherlands/219/2003(H7N7) and A/Zhejiang/DTID-ZJU01/2013(H7N9) viruses were purchased from Sino Biological Inc. HAs of the A/Washington/05/2011(H1N1) and A/California/04/2009(H1N1) viruses were prepared as described above. These HA proteins were used to detect their binding affinity (*K*_d_) for 3E1.

### Crystallization and structure determination

To prepare the 3E1 Fab–HA complex, the purified 3E1 Fab and HA were mixed in a 2:1 molar ratio (Fab:monomeric HA, six Fabs per HA trimer) and incubated overnight at 4 °C, and the excess Fab was removed by size exclusion chromatography with a Superdex 200 10/300 column. Crystallization was performed using the hanging drop vapour diffusion method at 20 °C by mixing an equal volume (0.1 μl) of the protein solution (10 mg ml^−1^) and the reservoir solution. Crystals of the 3E1 Fab–CA09 HA complex were grown from the drop with the reservoir solution containing 0.1 M MES (pH 6.5) and 16% (v/v) PEG 550 MME over two months. Crystals of the 3E1 Fab–WA11 HA complex were grown from the drop with the reservoir solution containing 0.1 M Na HEPES (pH 7.5), 20% (v/v) PEG 400 and 0.1 M LiCl over 4 months. Before collecting the diffraction data, the crystals were cryoprotected in the reservoir solution supplemented with 25% (v/v) glycerol, and then flash cooled in liquid nitrogen. The diffraction data were collected at −175 °C at BL19U1 of the National Facility for Protein Science in Shanghai and BL17U of the Shanghai Synchrotron Radiation Facility, and processed with HKL2000 (ref. 42).

The structure of the 3E1 Fab–CA09 HA complex was solved by the molecular replacement (MR) method, as implemented in Phaser of Phenix^43^ using the monomeric CA09 HA (PDB code 3LZG)^14^ and the daclizumab Fab (PDB code 3NFS)^44^ as the search models. The structure of the 3E1 Fab–WA11 HA complex was solved using the 3E1 Fab–CA09 HA complex as the search model. Structural refinement was performed with Phenix^43^ and Refmac5 (ref. 45), and the models were constructed using Coot^46^. The stereochemistry and quality of the structural models were analysed using Molprobity^47^, the PISA server^48^ and programs in the CCP4 suite and were validated using the wwPDB validation server (http://wwpdb-validation.wwpdb.org/validservice). All structures were generated using PyMOL (http://www.pymol.org). The diffraction data and structural refinement statistics are summarized in Table 1.

### Analysis of the epitope sequence

All full-length, non-identical influenza HA sequences (H1, H2, H5, H9, H13 and H16) derived from human hosts were downloaded from the NCBI Influenza Virus Database. The sequences were aligned using ClustalX (ref. 49), and the quality scores of each column show the conservation rates for each residue.

### Binding affinity (*K*_d_) assay

The binding affinity (*K*_d_) of the HAs from different virus strains for the 3E1 mAb was determined by bio-layer interferometry at 25 °C using an Octet Red instrument (ForteBio, Inc.). 3E1 mAb was immobilized on an anti-human IgG-Fc (AHC)-coated biosensor surface and then exposed to the different HAs in solution. 3E1 mAb was loaded onto an AHC-coated biosensor in kinetics buffer (1 × PBS, pH 7.4) for 300 s. To measure *k*_on_, the association of HAs was measured for 900 s by exposing the sensor to HAs in kinetics buffer. To measure *k*_off_, the dissociation of HAs was measured for 900 s in kinetics buffer. The *K*_d_ is represented by the *k*_off_/*k*_on_ ratio.

### Microneutralization assay

The microneutralization assay was performed as previously described^50^. Briefly, MDCK cells (ATCC) were maintained in Dulbecco’s Modified Eagle’s Medium supplemented with 10% fetal bovine serum at 37 °C. On the day of the experiment, MDCK cells grown in 96-well plates were washed twice with PBS and incubated in 100 μl per well Dulbecco’s Modified Eagle’s Medium (2% BSA) supplemented with 2 μg ml^−1^ trypsin-EDTA. 3E1 (100 μg ml^−1^) was serially diluted two-fold in 50 μl and then mixed with influenza virus (100 TCID_50_ in 50 μl per well). Positive control wells (virus only) and negative control wells (without virus) were included on each plate. After 1 h incubation at 37 °C in a 5% CO_2_ humidified atmosphere, the mixture was added to confluent MDCK monolayers. The cells were cultured for 24 h before the examined NP viral protein was detected with an ELISA using a polyclonal antibody against the influenza A NP protein, as previously described^50^. The neutralizing end point was assessed as previously described^50^. The inhibition ratio (%) was calculated as (OD (Pos. Control)−OD (Sample))/(OD (Pos. Control)−OD (Neg. Control)) × 100%.

### Prophylactic and therapeutic efficacy studies in mice

Six- to eight-week-old female SPF BALB/c mice were used in the *in vivo* experiments. In the prophylactic studies, groups of 5 mice each received a dose of 1, 3, 10 or 30 mg kg^−1^ of 3E1 or PBS buffer in a volume of 200 μl one day prior to intranasal challenge with 3 MLD_50_ of either A/Sichuan/1/2009(H1N1) or A/CK/NX/S6/2014(H5N6) virus. In the therapeutic studies, groups of 5 mice each received 20 mg kg^−1^ of 3E1 right after or 1, 2 or 3 days after challenge with either 3 MLD_50_ of the A/Sichuan/1/2009(H1N1) or A/CK/NX/S6/2014(H5N6) virus. PBS buffer was administered at day 1 post-challenge. The survival rates and weight-loss statuses of the mice were monitored until 14 days after infection. Animal care and use were in compliance with the guidelines of Institut Pasteur of Shanghai, SIBS, CAS. The *in vivo* experiments with the H5N6 virus were conducted in the enhanced animal biosafety level 3 (ABSL3^+^) facility at Harbin Veterinary Research Institute of the Chinese Academy of Agricultural Sciences after receiving approval from the Ministry of Agriculture of China and the China National Accreditation Service for Conformity Assessment.

### *In vitro* binding assay

The full-length, wild-type HA from A/Washington/05/2011 (H1N1) (WA11) was inserted into the pBudCE4.1 vector. The constructs containing mutants were generated by PCR and verified by DNA sequencing. To express the wild-type and mutant HAs, the HEK-293 T cells were plated in 6-well plates and individually transfected with these constructs. At 48–72 h after transfection, the cells were harvested and lysed with RIPA buffer. The detergent dodecylmaltoside was added to a final concentration of 0.5% to protect the transmembrane domain of HA, enabling HA to maintain its natural conformation. Then, an ELISA was performed to quantify the HAs in the supernatant of the lysates. In detail, at a concentration of 10 μg ml^−1^ per well, a mouse mAb named S-95-7, which targets the head region of HA, was added to the supernatant of the lysate to bind to the HAs. Subsequently, the rabbit anti-mouse serum was added to bind to S-95-7. Thus, using the OD_450_ of the ELISA, we determined the relative amount of HAs in the supernatants of the lysates. Then, another ELISA was performed to analyse the relative binding affinities of these mutant HAs with 3E1. In detail, the supernatants of the lysates containing the same amount of wild-type or mutant HAs were incubated with 3E1 mAb. We obtained different OD_450_ values, reflecting the different binding affinities of the HAs for 3E1. Finally, the relative binding affinities were reflected by the binding ratio, which was represented by the OD_450-mutations_/OD_450-wild type_ ratio.

### Cell-cell fusion inhibition assay

The HEK-293 T cells were transfected with the pBudCE4.1 plasmid (Invitrogen) containing the DNA fragment encoding HA0 of the A/Washington/05/2011(H1N1) strain. The cells were cultured in 6-well plates and transfected with the plasmids using the Lipofectamine transfection reagent (Invitrogen). At 24 h after transfection, the cells were treated with TPCK-treated trypsin (2.5 μg ml^−1^) for 10 min at 37 °C to cleave HA0 into HA1 and HA2. Afterwards, the cells were washed with PBS to decrease the pH to 5.0. Syncytia formation was apparent. To investigate the neutralizing mechanism of 3E1, the cells were incubated with 100 μg ml^−1^ 3E1 or 7F2 (neutralizing antibody against HCV) for 30 min at 37 °C after the trypsin digestion, respectively, and then fixed with 4% (w/v) paraformaldehyde and stained with 1% (w/v) toluidine blue in PBS. The cells were observed with an optical microscope and imaged. The experiment was replicated three times at least.

### Protease susceptibility assay

A total of 40 μg of the recombinant trimeric HA0 from the WA11 virus was digested to HA1 and HA2 with TPCK-treated trypsin (200:1 ratio by mass). The protease inhibitor (Aprotinin, Sigma-Aldrich) was added to stop the reaction and then excluded by buffer exchange using a desalting column. Next, the protein was incubated either with 3E1 or a control mAb (7F2) (1:2 ratio by molar) for 2 h at 37 °C, besides another control without mAb. Then, the pH was lowered to 5.0 by replacing the buffer with 100 mM sodium acetate in all samples except for the controls. Being thoroughly mixed, the samples were incubated for 30 min at 37 °C. After incubation, the samples was equilibrated at room temperature, and the pH was neutralized by addition of 200 mM Tris (pH 8.5). Then, TPCK-treated trypsin (1:150 ratio by mass) was added and all samples were incubated at 37 °C for 30 min. The reaction was quenched by the addition of non-reducing SDS–polyacrylamide gel electrophoresis (SDS–PAGE) loading buffer and boiled for 10 min. The samples were analysed by SDS–PAGE. The experiment was replicated three times at least.

### Data availability

The full-length influenza A virus HA sequences were downloaded from the Influenza Virus Resource at the National Center for Biotechnology Information (NCBI) database and the Global Initiative on Sharing All Influenza Data (GISAID) database. The sequences used in Fig. 1a were: H1 A/Brevig Mission/1/1918 (UniProtKB: Q9WFX3.2), H1 A/WSN/1933 (GenBank: ACF54598.1), H1 A/California/07/2009 (NCBI Reference Sequence: YP_009118626.1), H1 A/Washington/05/2011 (GenBank: AGK72598.1), H1 A/Brisbane/59/2007 (GenBank: ACA28844.1), H5 A/Hong Kong/483/1997 (GenBank: ACZ48509.1), H5 A/Vietnam/1203/2004 (GenBank: ABW90135.1), H5 A/Anhui/1/2005 (GenBank: ADG59080.1), H5 A/Sichuan/26221/2014 (GISAID Accession: EPI533583), H3 A/Hong Kong/1968 (GenBank: AFG71887.1), H3 A/Aichi/1968 (GenBank: BAN81712.1), H2 A/Canada/720/2005 (GenBank: AAY28987.1), H9 A/Hong Kong/1073/99 (NCBI Reference Sequence: NP_859037.1), H13 A/black-headed gull/Netherlands/1/00 (GenBank: AAV91212.1), H16 A/black-headed gull/Sweden/5/99 (GenBank: AAV91217.1), H3 A/Brisbane/10/2007 (GenBank: ABW23353.1), H3 A/Taiwan/70113/2007 (GenBank: ACQ65702.1), H7 A/Netherlands/219/2003 (GenBank: AAR02640.1), H7 A/Shanghai/02/2013 (NCBI Reference Sequence: YP_009118475.1). The crystal structures of the 3E1 Fab–CA09 HA complex and the 3E1 Fab–WA11 HA complex have been deposited in the Protein Data Bank under accession codes 5GJS and 5GJT, respectively. The amino acid sequence of 3E1 Fab has also been deposited in the Protein Data Bank. The authors declare that all other data supporting the findings of this study are available within the article and its Supplementary Information Files.

## Additional information

**How to cite this article:** Wang, W. *et al*. Human antibody 3E1 targets the HA stem region of H1N1 and H5N6 influenza A viruses. *Nat. Commun.* **7,** 13577 doi: 10.1038/ncomms13577 (2016).

**Publisher’s note**: Springer Nature remains neutral with regard to jurisdictional claims in published maps and institutional affiliations.

## Supplementary Material

Supplementary Information Supplementary Figures 1-6

Peer Review

## Figures and Tables

**Table 1:** Statistics of X-ray diffraction data and structure refinement.

	**3E1 Fab-CA09HA**	**3E1 Fab-WA11HA**
*Diffraction data*		
Space group	*H32*	*H32*
Cell dimensions		
*a=b* (Å)	130.2	130.8
*c* (Å)	365.9	366.9
Resolution (Å)	50.0–2.90 (3.00–2.90)*	50.0–3.10 (3.21–3.10)*
Observed reflections	162,952	283,408
Unique reflections (*I*/σ(*I*)>0)	25,201	22,417
Average*I*/σ(*I)*	12.9 (2.6)*	16.7 (3.9)*
Completeness (%)	93.8 (100.0)*	100.0 (100.0)*
Redundancy	6.5 (6.3)*	12.6 (13.1)*
*R*merge (%)†	16.7 (80.3)*	10.5 (47.7)*
*R*pim (%)‡	5.7 (29.6)*	3.1 (13.6)*
CC1/2 (%)	99.4 (72.6)*	99.5 (95.6)*
		
*Refinement and structure model*		
Resolution (Å)	47.98–2.90	37.76–3.10
No. reflections	23,695	21,207
Working set	22,518	20,122
Test set	1,177	1,085
R_work_/R_free_ (%)§	25.1/30.4	21.6/25.9
No. atoms		
Protein	7,028	7,069
Carbon hydrate	42	28
Water	0	0
Wilson B-factor (Å^2^)	47	63
Average B-factors (Å^2^)	52	71
Protein	52	71
Carbohydrate	73	86
Water	0	0
RMS deviations		
Bond lengths (Å)	0.011	0.011
Bond angles (^o^)	1.4	1.3
Ramachandran plot (%)		
Favored	95.0	95.0
Allowed	5.0	4.9
Outliers	0.0	0.1

^*^Numbers in parentheses represent the highest resolution shell.

^†^Rmerge==_hkl_=_i_|I_i_(hkl)−<I(hkl)>|/=_hkl_=_i_I_i_(hkl).

^‡^Rpim=Σ_hkl_(1/(n-1))^1/2^Σ_i_|I_i_(hkl)−<I(hkl)>|/Σ_hkl_Σ_i_I_i_(hkl).

^§^R==_hkl_||F_o_|−|F_c_||/=_hkl_|F_o_|.

**Table 2:** Sequence identity of 3E1 epitope by subtype.

**Residue**		Consensus*	**CA04/H1 Sequence**	**Per cent Identity by Subtype**					
				**H1 (5532)**†	**H5 (217)**†	**H2 (80)**†	**H9 (6)**†	**H13 (93)**‡	**H16 (38)**‡
HA1	18	H	H	99	100	100	0 (Q)§	0 (L)§	0 (L)§
	38	H	H	99	99	100	100	0 (S)§	0 (S)§
	326	T	T	99	100	100	78 (V)§22 (I)	100	100
HA2	16	G	G	99	100	100	100	100	100
	18	V	V	97	100	97	100	98 (I)§	100 (I)§
	19	D	D	99	100	100	0 (A)§	0 (N)§	0(N)§
	20	G	G	100	100	100	100	100	100
	21	W	W	100	100	100	100	100	100
	34	Y	Y	99	100	100	0(M)§	26(I)§	0(I)§
	38	L/K	L	88 (L)§12 (Q)	98(K)§	100(K)§	100(R)§	100(K)§	100(K)§
	42	Q	Q	100	100	100	100	100.0	100
	45	I	I	99	99	0(F)§	78 (I)§22(V)	100	96
	48	I	I	99	0(V)§	100	100	100	100
	49	T	T	99	100	100	100	100	100
	52	V	V	99	100	90	100	0(I)§	0(I)§

^*^Most common residue at position by simple majority across all group 1 sequences. HA1 residues are listed first, followed by HA2 residues.

^†^Number of sequences available for subtype infecting humans at time of download.

^‡^Number of sequences available for subtype at time of download.

^§^Most common residue in this subtype.
