# Supplementary material for: Human antibody 3E1 targets the HA stem region of H1N1 and H5N6 influenza A viruses
Source: Nat Commun. 2016 Dec 2;7:13577. doi: 10.1038/ncomms13577 (PMC5477490; doi:10.1038/ncomms13577)
Supplement: Supplementary Information — Supplementary Figures 1-6 [file ncomms13577-s1.pdf]

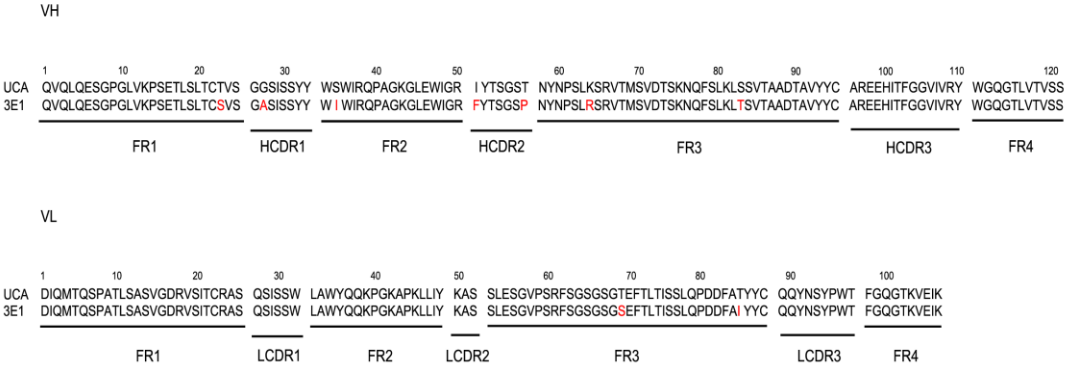

Supplementary Figure 1. Alignment of VH and VL amino acid sequences of 3E1 and its unmutated common ancestor (UCA). The amino acid residues are numbered and the CDR and FR segments are labeled. The amino acid substitutions are highlighted in red.

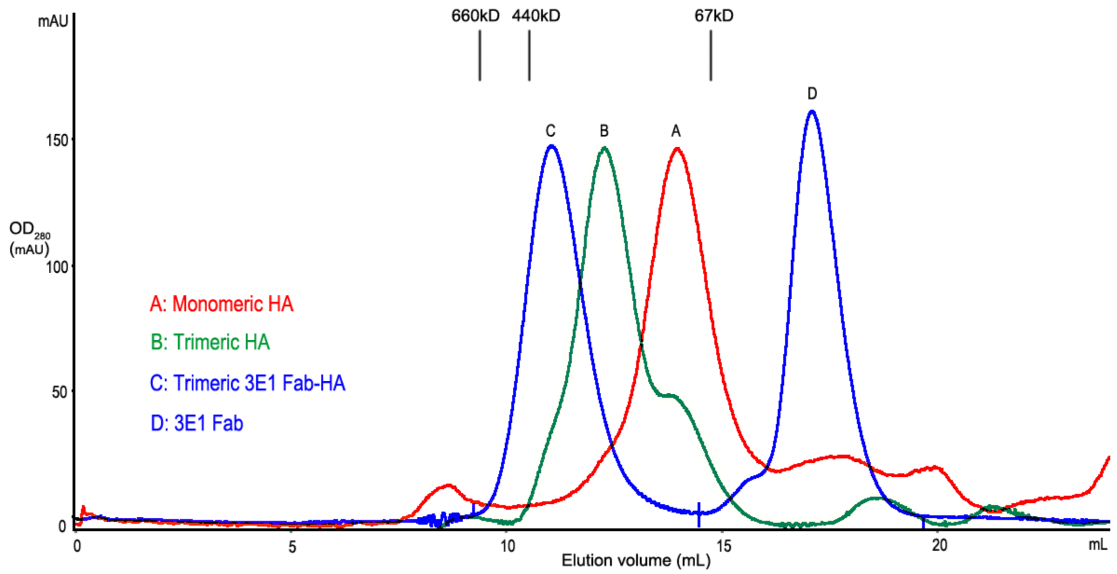

Supplementary Figure 2. Gel filtration analysis of CA09 HA. The wild-type CA09 HA has an elution peak at 13.97 ml, corresponding to an apparent molecular weight of ~70 kDa, indicating that it exists as a monomer (theoretical molecular weight of 57 kDa) in solution. The CA09 HA mutant containing the G205C and R220C mutations that form a disulfide bond at the monomer-monomer interface, has an elution peak at 12.24 ml, corresponding to an apparent molecular weight of ~200 kDa, indicating that it exists as a trimer (theoretical molecular weight of 171 kDa). The trimeric 3E1 Fab-CA09 HA complex has an elution peak at 11.03 ml, corresponding to an apparent molecular weight of ~400 kDa (theoretical molecular weight of 312 kDa). The excessive 3E1 Fab in the complex solution has an elution peak at 17.08 ml.

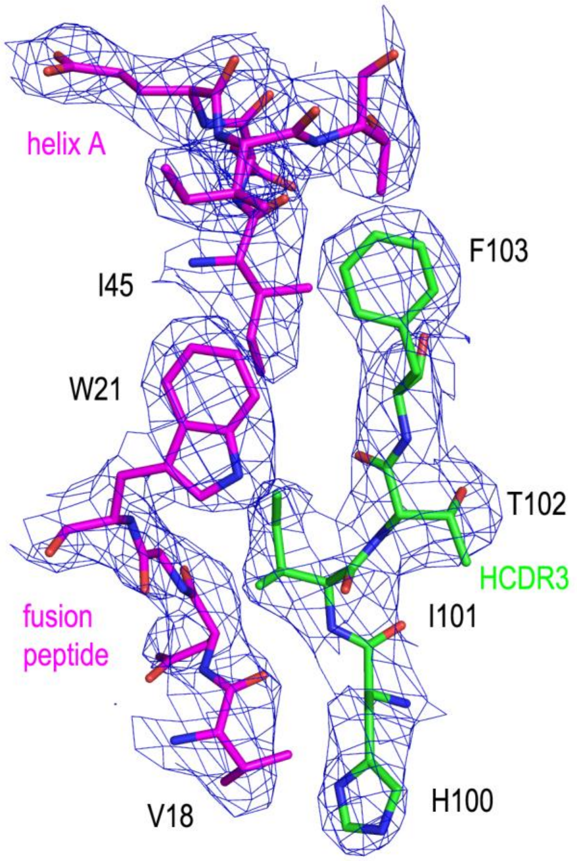

Supplementary Figure 3. Representative 2Fo-Fc map showing a portion of the interaction interface between the 3E1 Fab and the CA09 HA. The map is contoured at 1.0σ level with the final structure shown in stick models.

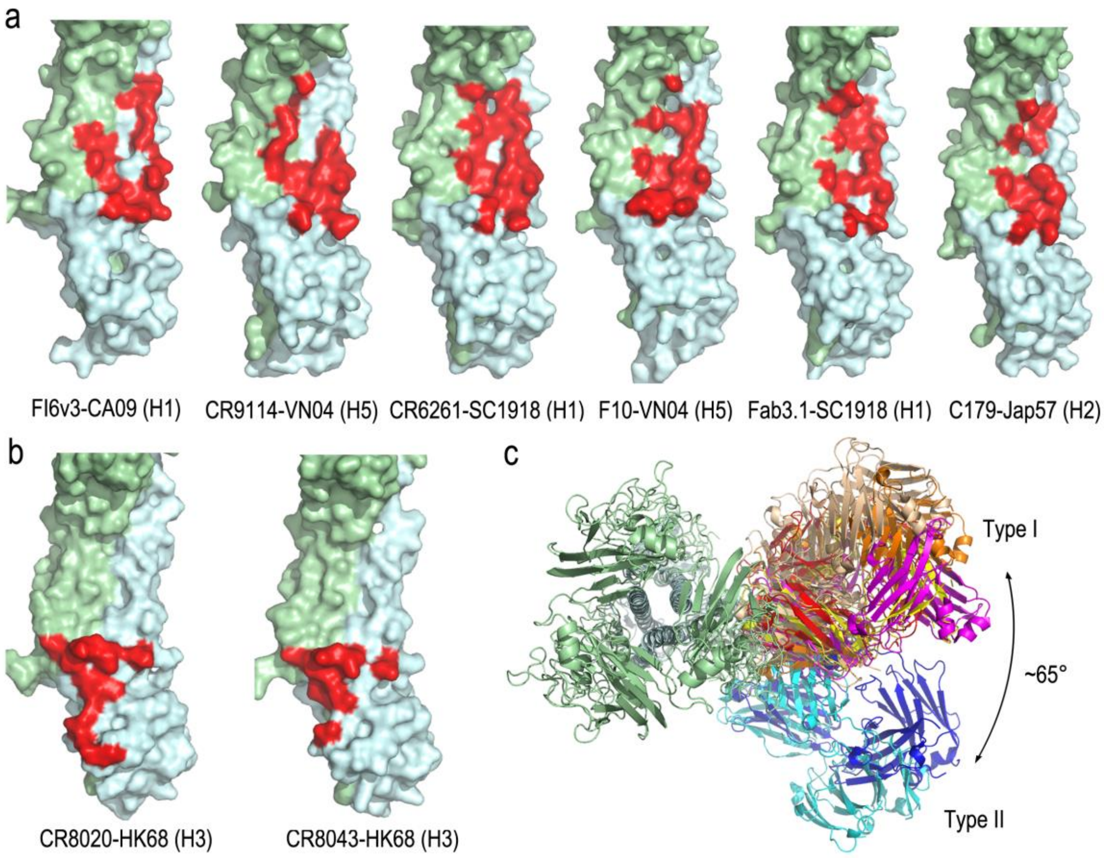

Supplementary Figure 4. Two types of bnmAbs target different epitopes on the stem region of HAs with different orientations. (a) Type I bnmAbs target the F subdomain. One monomeric HA is shown in surface representation, with HA1 in pale green, HA2 in pale cyan, and the epitope in red. **(b)** Type II bnmAbs target epitopes composed of the fusion peptide and the outermost β-strand preceding helix A. **(c)** The type I and type II bnmAbs bind to HAs from different orientations. For clarity, only one HA trimer is shown in cartoon. The Fabs of FI6v3, CR9114, CR6261, F10, Fab3.1 and C179 of type I bnmAbs are shown in red, magenta, yellow, light orange, wheat, and orange, respectively, and the Fabs of CR8020 and CR8043 of type II bnmAbs in blue and cyan, respectively. The binding orientations of the type I and type II bnmAbs differ by about 65°.

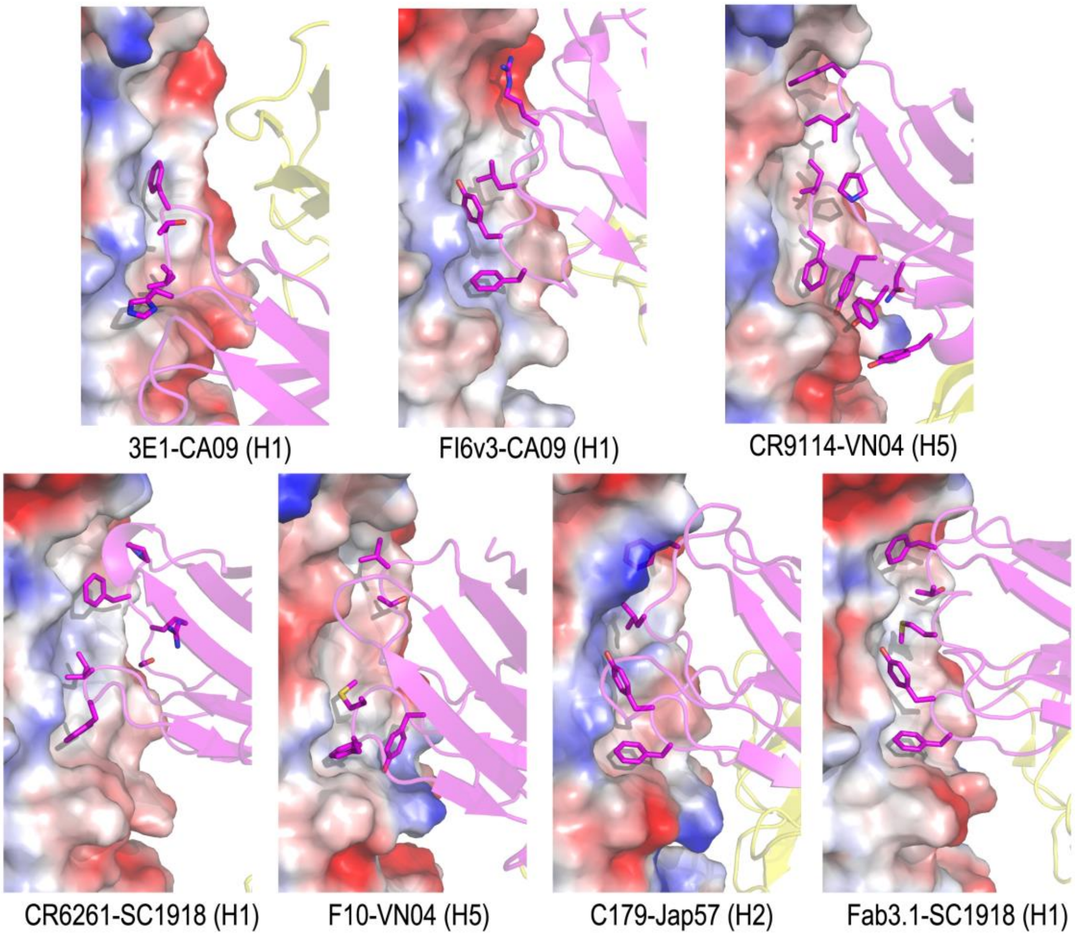

Supplementary Figure 5. Fabs of type I bnmAbs make hydrophobic contacts with the F subdomain of HA. The HAs are shown as electrostatic surface representations and the Fabs as cartoons in the same orientation. The residues of Fabs involved in the hydrophobic contacts are shown with side chains.

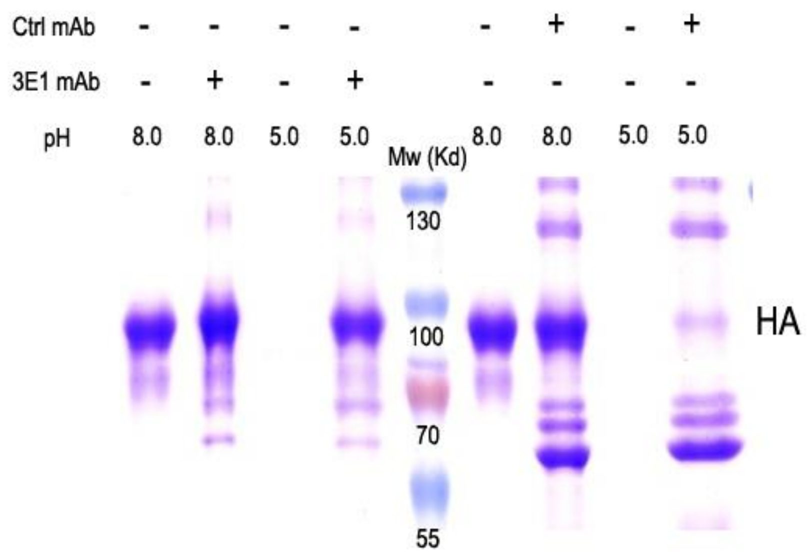

Supplementary Figure 6. Protease sensitivity assay of HA in the presence of 3E1 mAb. Exposure of HA to low pH converts the HA to the protease-susceptible, post-fusion state (lanes 3, 8). Treatment of HA with 3E1 mAb before low-pH treatment, but not the control mAb, blocks the pH-induced conformational change, retaining HA in the protease-resistant, pro-fusion state (lanes 4, 9). Data represent a representative experiment from three independent experiments.
