# Supplementary material for: Human antibody 3E1 targets the HA stem region of H1N1 and H5N6 influenza A viruses
Source: Nat Commun. 2016 Dec 2;7:13577. doi: 10.1038/ncomms13577 (PMC5477490; doi:10.1038/ncomms13577)
Supplement: Peer Review [file ncomms13577-s2.pdf]

Editorial Note: This manuscript has been previously reviewed at another journal that is not operating a transparent peer review scheme. This document only contains reviewer comments and rebuttal letters for versions considered at Nature Communications.

PEER REVIEW FILE

Reviewers' Comments:

Reviewer #2 (Remarks to the Author):

The authors describe here functionally and structurally a previously published mAb able to target the HA stem region of a range of H1 and H5 influenza A viruses. They show protective and limited therapeutic action against an H1 and an H5 strain in vivo in mice, and go on to determine in detail the structural characteristics of HA binding by the mAb. The authors conclude that the HA epitope recognised here comprises parts of the two epitope clusters previously described for stem-specific mAbs and consequently, that this mAb binds with a different geometry compared to the two described broadly neutralising mAb classes. Mutation analysis was performed to establish why other HA subtypes are not recognised by the mAb. This is a solid and detailed structural study. Given the fast-moving field of broadly binding mAbs against influenza HA, the degree of novelty of the study may however not merit publication in Nature Communications.

Major concern:
1. Fig. shows minimal differences in weight loss, which translate into major differences in mortality. This indicates a cut-off for mortality that is right where the maximum weight loss sits and therefore, mortality curves suggests a greater effect of this mAb than it really has in terms of reducing morbidity.

Reviewer #3 (Remarks to the Author):

This is a comprehensive study of human neutralizing antibody 3E1 against H1 and H5 subtype influenza viruses. The manuscript was well written, and the author responses to referee's comments (Reviewer #1) are all appropriate.

Additional comments to improve the figure quality for publication:
Figures 3 & 4 need to be revised to improve the figure clarity especially the coloring in Figure 3b and Figure 4a. In Figure 3b, the antibody heavy and light chain should be colored in light colors

as background, so the epitope residue side-chains and the paratope regions can be shown clearly. In Figure 4a, three Fab models, FI6v3, 3E1 and CR8020, should be highlighted in different colors to differentiate.

The ray shadow from PyMOL could be removed to make Figures 3 & 4 more clear.

Reviewer #2

Comments (Major concern): Fig. shows minimal differences in weight loss, which translate into major differences in mortality. This indicates a cut-off for mortality that is right where the maximum weight loss sits and therefore, mortality curves suggests a greater effect of this mAb than it really has in terms of reducing morbidity.

Answer: We appreciate the criticism by the reviewer; however, we believe that the weight loss curves and mortality curves of 3E1 are consistent with each other and thus are acceptable.

First, the similar observations have been reported when the mice were treated with 9H10, CT149 or CR8043 antibodies, the weight loss curves showed a minimal differences, but still the differences of mortality were observed in influenza virus infected mice[1-3].

Second, in the H1 therapy experiments, although the maximum weight loss in some mice reached to the cut-off, later on some of the mice treated with 3E1 recovered and survived from the H1 influenza virus infection. Consistently, this protective efficacy correlated with time point treated with 3E1 (1 d.p.i 80% vs 2 d.p.i 20%). From this point of view, 3E1 is effective against H1N1 virus.

Reviewer #3

Comments: Figures 3 & 4 need to be revised to improve the figure clarity especially the coloring in Figure 3b and Figure 4a. In Figure 3b, the antibody heavy and light chain should be colored in light colors as background, so the epitope residue side-chains and the paratope regions can be shown clearly. In Figure 4a, three Fab models, FI6v3, 3E1 and CR8020, should be highlighted in different colors to differentiate. The ray shadow from PyMOL could be removed to make Figures 3 & 4 clearer.

Answer: We are grateful to the constructive suggestions. We have changed the color coding of Figures 3 and 4 according to the reviewer’s suggestions: the heavy and light chains of the antibodies are colored in light colors (heavy chain in violet, light chain in yellow) as the background and the residues of the epitope and paratope regions can be shown clearly.

In Figure 4a, we have also used three distinctive colors to represent the Fab models of FI6v3 (green), 3E1 (orange) and CR8020 (red).

We have also removed the ray shadow in Figures 3 and 4 to make them clearer.

1 Tan, G. S. *et al.* Characterization of a broadly neutralizing monoclonal antibody that targets the fusion domain of group 2 influenza A virus hemagglutinin. *Journal of virology* **88**, 13580-13592, doi:10.1128/JVI.02289-14 (2014).

2 Wu, Y. *et al.* A potent broad-spectrum protective human monoclonal antibody crosslinking two haemagglutinin monomers of influenza A virus. *Nature communications* **6**, 7708, doi:10.1038/ncomms8708 (2015).

3 Friesen, R. H. *et al.* A common solution to group 2 influenza virus neutralization. *Proceedings of the National Academy of Sciences of the United States of America* **111**, 445-450, doi:10.1073/pnas.1319058110 (2014).
